# Artificial Intelligence Applied to Pancreatic Imaging: A Narrative Review

**DOI:** 10.3390/healthcare10081511

**Published:** 2022-08-11

**Authors:** Maria Elena Laino, Angela Ammirabile, Ludovica Lofino, Lorenzo Mannelli, Francesco Fiz, Marco Francone, Arturo Chiti, Luca Saba, Matteo Agostino Orlandi, Victor Savevski

**Affiliations:** 1 Artificial Intelligence Center, IRCCS Humanitas Research Hospital, Via Manzoni 56, Rozzano, 20089 Milan, Italy; 2 Department of Biomedical Sciences, Humanitas University, Via Rita Levi Montalcini 4, Pieve Emanuele, 20072 Milan, Italy; 3 Department of Diagnostic and Interventional Radiology, IRCCS Humanitas Research Hospital, Via Manzoni 56, Rozzano, 20089 Milan, Italy; 4 Department of Radiology, SDN, 80131 Napoli, Italy; 5 Nuclear Medicine Unit, Department of Diagnostic Imaging, E.O. Ospedali Galliera, 56321 Genoa, Italy; 6 Department of Nuclear Medicine and Clinical Molecular Imaging, University Hospital, 72074 Tübingen, Germany; 7 Department of Nuclear Medicine, IRCCS Humanitas Research Hospital, Via Manzoni 56, Rozzano, 20089 Milan, Italy; 8 Department of Radiology, University of Cagliari, 09124 Cagliari, Italy; 9 Department of Radiology, ASST Lodi, Ospedale Maggiore di Lodi, 26900 Lodi, Italy

**Keywords:** artificial intelligence, radiomics, pancreatic imaging, MRI, CT, PET

## Abstract

The diagnosis, evaluation, and treatment planning of pancreatic pathologies usually require the combined use of different imaging modalities, mainly, computed tomography (CT), magnetic resonance imaging (MRI), and positron emission tomography (PET). Artificial intelligence (AI) has the potential to transform the clinical practice of medical imaging and has been applied to various radiological techniques for different purposes, such as segmentation, lesion detection, characterization, risk stratification, or prediction of response to treatments. The aim of the present narrative review is to assess the available literature on the role of AI applied to pancreatic imaging. Up to now, the use of computer-aided diagnosis (CAD) and radiomics in pancreatic imaging has proven to be useful for both non-oncological and oncological purposes and represents a promising tool for personalized approaches to patients. Although great developments have occurred in recent years, it is important to address the obstacles that still need to be overcome before these technologies can be implemented into our clinical routine, mainly considering the heterogeneity among studies.

## 1. Introduction

Pancreatic imaging is one of the body imaging domains that has witnessed increasing interest from researchers due to challenging differential diagnosis and high morbidity. Both computed tomography (CT) and magnetic resonance imaging (MRI) have been widely used as essential tools in the early diagnosis and staging of pancreatic disease [1]. The diagnosis of pancreatic pathologies usually requires the combined use of different morphological and functional imaging modalities, mainly, CT, MRI, and positron emission tomography (PET). Native CT and contrast-enhanced CT (CECT) are widely used for the diagnosis of pancreatic pathologies but also for presurgical evaluation, as they help to determine tumor size, evaluate vascular involvement, and identify disease spread. MRI is useful for characterizing both cystic lesions and solid tumors as it allows noninvasive evaluation of the pancreatic ducts, pancreatic parenchyma, adjacent soft tissues, and vascular network [2,3]. PET utilizes many radiopharmaceuticals to characterize biological features of tumors such as metabolic intensity and receptor expression [4,5].

Artificial intelligence (AI) has the potential to transform the clinical practice of medical imaging due to its ability to discriminate subtle image features. For this reason, it has recently emerged as a noninvasive tool for a better characterization of lesions, thus helping to achieve a more “personalized” approach [5,6].

Computer-aided diagnosis (CAD) and radiomics have been applied to various radiological techniques for different purposes, such as segmentation, detection, and characterization of lesions, risk stratification, and prediction of response to treatments [7,8].

However, their potential applications in pancreatic imaging are still under investigation, both for non-oncological and oncological purposes.

The aim of the present review is to critically assess the available literature on the role of AI and radiomics applied to pancreatic imaging.

## 2. Materials and Methods

We performed a search of PubMed, Scopus, Embase, Web of Science, and Cochrane library databases for articles relevant to the application of AI and radiomics in pancreatic imaging. On the PubMed database, we used the following MeSH headings: “artificial intelligence”, “Machine Learning”, “Deep Learning”, “neural networks, computer”. The search algorithm was constructed using specific strings for each library, as reported in Supplementary Material File S1.

We included original research papers based on human subjects, written and published (including those distributed as “online first”) in the English language up to July 2021 that focused on CT, MRI, or PET as imaging techniques. 

**Figure 1:**
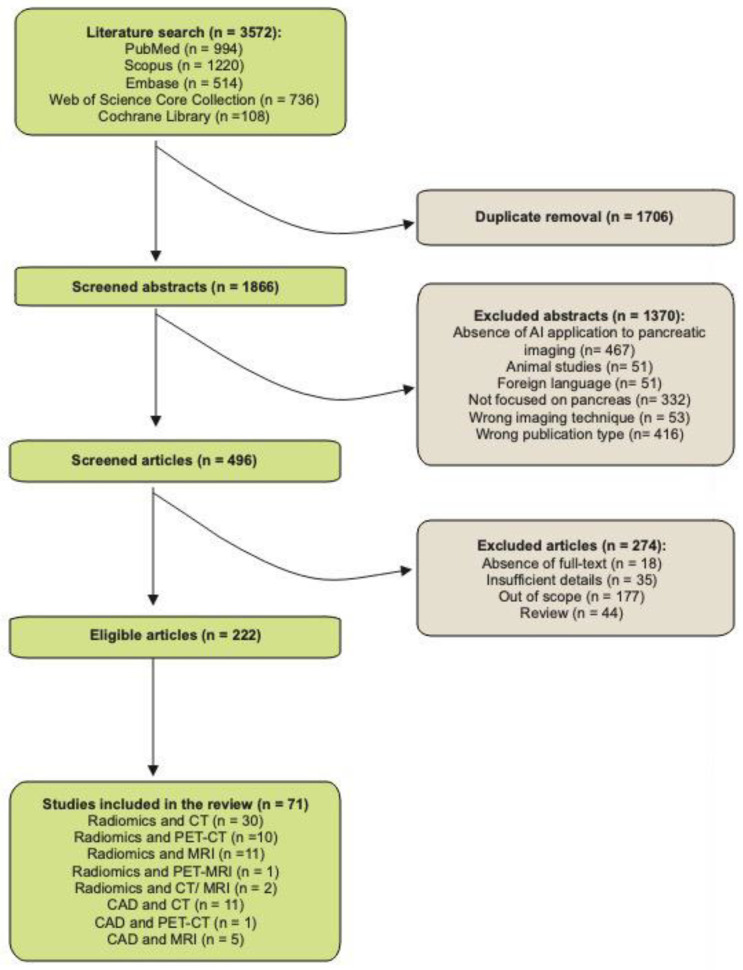
Flowchart of the study inclusion process.

After screening for duplicates and eligibility, we further refined the selection of manuscripts according to our subjective assessment of their relevance and novelty. The detailed description of the study inclusion process is reported in Figure 1.

## 3. Insights on Radiomics Applied to Pancreatic Imaging

**Figure 2:**
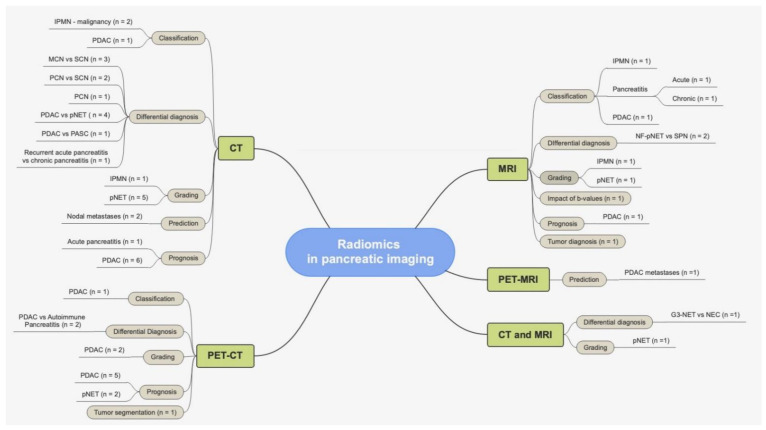
Summary of radiomics applications in pancreatic imaging.

In recent years, researchers have investigated the field of radiomics to try and improve patient care [7]. This is even more compelling when dealing with pancreatic imaging, as both clinicians and surgeons have been relying on imaging to improve work-up and prognosis in a field where mortality and morbidity are still very high [2,3] (Figure 2). 

### 3.1. Radiomics and CT

CT represents, by far, the most diffused multislice diagnostic method, with an estimated 400 million examinations per year worldwide. This availability of data has made large-scale radiomics studies possible. In pancreatic imaging, CT features both an excellent spatial and temporal resolution, allowing for a precise assessment of small structures as well as enabling the evaluation of multiple contrast phases. These characteristics have made it possible for CT to be used in a variety of oncological and non-oncological settings [9,10,11,12,13,14,15,16,17,18,19,20,21,22,23,24,25,26,27,28,29,30,31,32,33,34,35,36,37].

#### 3.1.1. Oncological Applications

Differentiating various lesions (from benign to different levels of malignancy) is the foundation of patient care assessment and many authors have been extensively studying this field. One of the most frequent differential diagnoses a radiologist can come across is differentiating cystic lesions. While some authors have tried to create an algorithm that ties together clinical and radiological features with radiomics signatures [11,13,14], others focus solely on radiomics and its validity in differentiating different cystic neoplasms [9,10,12]

When investigating pancreatic lesions, pancreatic neuroendocrine tumors (pNETs) have a peculiar enhancement pattern that differentiates them from other tumors. Nevertheless, when coming across very small or heterogeneous lesions, it is still quite challenging to understand whether it is a pNET or a pancreatic ductal adenocarcinoma (PDAC); hence, the majority of research is found in this field. We found that most authors focus on differentiating a PDAC from pNET [19,20,21,23], whereas others investigated radiomics use to predict the histological grade of a pNET [22,25,26,27].

On the other hand, when it comes to adenocarcinoma, the core investigation is about prognosis prediction. Recent studies show how radiomics features could be related with mortality, whether based on tumor analysis [32,33,34] or node involvement [35,36].

##### Cystic Lesions

Cystic findings within the pancreatic parenchyma represent a diagnostic challenge since it might be hard to tell apart pure benign lesions from the ones with transformation potential. However, the combined evaluation of macroscopic morphological features and texture analysis has afforded a great improvement in the diagnostic performance when it comes to the characterization of cystic lesions [9].

Yang et al. [9] evaluated the consistency of textural features between different slice thicknesses extracted from CT images of 2 mm- and 5 mm-thick slices and found a good correlation among the extracted features. They included 25 patients with mucinous cystic neoplasms (MCNs) and 53 patients with serous cystic neoplasms (SCNs) using a preliminary model based on texture extracted from CECT images selected via LASSO regression and random forest classifiers. In the validation group, they achieved an accuracy of 74% in the 2 mm slice-thickness group and 83% in the 5 mm slice-thickness group. This study highlighted the ability of radiomics in reducing misdiagnosis and avoiding overtreatment. In a similar study, the same authors assessed the potential of CT texture analysis in discriminating pancreatic serous cystadenoma from mucinous cystadenoma and improving the diagnostic performance by combining morphological characteristics and textural features. They used a combination of morphological characteristics of CT images and textural features that achieved an impressive AUC of 0.893 [10].

Shen et al. [11] analyzed the potential of CECT in the discrimination of the different subtypes of pancreatic cystic neoplasms (PCNs), considering that only SCNs have a lower malignant potential and need only periodic follow-up. Using a Boruta algorithm, five radiomics features (Histogram_Entropy, Histogram_Skeweness, LLL_GLSZM_GLV, Histogram_Uniformity, HHL_Histogram_Kurtosis) and four clinical factors (serum carbohydrate antigen 19-9, sex, age, and serum carcinoembryonic antigen) were significantly different across SCNs, MCNs and intraductal papillary mucinous neoplasms (IPMNs). Among the three machine learning (ML) algorithms, the random forest classifier achieved an accuracy of 79.59% in the validation cohort.

In a different study, Wei et al. [12] also used radiomics as a way to avoid overtreatment of benign lesions, as they used it as a diagnostic model to differentiate SCNs from other PCNs. Their results showed that texture features, including intensity T-range, wavelet intensity T-median, and wavelet neighborhood gray-tone difference matrix (NGTDM) busyness, and five guideline-based features (sex, location, moment difference, mean rectangular fitting factor, and size) were the most statistically significant in identifying SCNs. The model achieved an accuracy of ~76% in the cross-validation cohort and ~83% in an independent validation cohort.

Xie et al. [13] applied radiomics to differentiate atypical serous cystadenomas (ASCNs) and MCNs and compared radiomics and radiological analysis. They trained an ML model with radiomics features and then demonstrated that adding radiological features such as lesion location, shape, cyst wall, and wall enhancement into this model could significantly improve its performance. Similarly, Tobaly et al. [14] developed a radiomics model mostly based on high-order CT radiomics features, which showed high diagnostic performance in differentiating benign from malignant IPMNs. Their results showed that 85 radiomics features were significantly different between patients with benign and malignant IPMNs, reaching an AUC of 0.84.

Recently, Chen et al. [15] also developed and validated a CT-based radiomics nomogram for differentiating SCNs from mucin-producing PCNs in a preoperative setting. They included a total of 89 patients (31 SCNs, 30 IPMNs, and 28 MCNs) who underwent preoperative CT. The authors used a comprehensive nomogram incorporating clinical features and a fusion radiomics signature. The fusion radiomics signature used was obtained by the combination of the radiomics features extracted in the plain, late arterial, and venous phases. This nomogram obtained an AUC of 0.960 in the training cohort and 0.817 in the validation cohort.

Other studies focused on the prediction of the malignant potential of the IPMN whose management is challenging, owing to the low reliability of conventional imaging techniques in the identification of suspicious lesions. Hanania et al. [16] correlated the histopathological grade of an IPMN with a cross-validated panel of 10 radiomics markers within the cyst contours, reaching an AUC of 0.96 at a sensitivity of 97% and specificity of 88%. Permuth et al. [17] tested a combined model of radiomics features and miRNA genomic classifier (MGC) data, considered as potential biomarkers of pancreatic tumorigenesis, to achieve an AUC of 0.95, higher than those of the single variables (AUC of 0.77 for radiomics features and AUC of 0.83 for MGC data). Attiyeh et al. [18] focused only on a branch-duct IPMN (BD-IPMN) on a preoperative CT scan to create a combined model of quantitative and clinical features. It reached an AUC of 0.79, outperforming the single models (AUC of 0.67 for radiomics and AUC of 0.76 for clinical parameters), and the quantitative mural nodularity feature demonstrated a significant role in the prediction of high-risk disease.

##### pNET

For pNETs, the use of radiomics and CT has been studied for differentiating and classifying pancreatic tumors. He et al. [19] developed three models to differentiate nonfunctional pNETs and PDACs. Their model combined a radiomics signature and clinical-radiological features, reaching an AUC of 0.960 and 0.884 in the primary and validation cohort, respectively.

In relation to pNETs, Li et al. [20] examined the use of volumetric CT texture analysis in differentiating atypical pNETs from PDACs. The authors retrospectively analyzed 127 patients with 50 PDACs and 77 pNETs. They found that the fifth percentile and fifth+skewness were optimal parameters for alone and combined diagnosis with an AUC of 0.811 and 0.792, respectively, compared to the mean CT value (AUC = 0.678).

In another study by Yu et al. [21], radiomics was used to differentiate non-hypervascular pNETs from PDACs in 120 patients. Specifically, the authors compared the performances of significant features on conventional imaging techniques (maximum diameter on axial section, margins, calcification, tumor vascularity, and heterogeneity) and texture analysis both in the arterial phase and portal vein phase, achieving an AUC of 0.780, 0.855, and 0.929, respectively, on logistic regression.

Canellas et al. [22] also assessed whether CT texture and CT features are predictive of pNETs. Preoperative contrast-enhanced CT images of 101 patients with pNETs were assessed. The images were evaluated for tumor location, tumor size, tumor pattern, predominantly solid or cystic composition, presence of calcification, presence of heterogeneous enhancement on contrast-enhanced images, presence of pancreatic duct dilatation, presence of pancreatic atrophy, presence of vascular involvement by the tumor, and presence of lymphadenopathy. Their results showed that a size larger than 2.0 cm is useful for predicting aggressive tumors (grades 2 and 3). The only texture parameter predictive of tumor grade was entropy with a spatial scale filter 2 (AUC of 0.65).

Reinert et al. [23] also assessed the role of CT texture analysis for differentiation of PDACs from pNETs in the portal venous phase, comparing this data with visual assessment and tumor-to-pancreas attenuation ratios obtained by placing large hand-drawn ROIs in ^18^F-FDG PET/CT, 68Ga-DOMITATE-PET/CT, dual-phase CT (including an arterial phase), and contrast-enhanced MRI of the pancreas. They obtained highly significant (*p* < 0.005) discriminatory textural features between PDACs and pNETs, and their model correctly classified PDACs or pNETs in 75.8% of patients. Specifically, 42/53 patients were predicted correctly as PDACs (sensitivity 79.2%) and 12/42 patients were predicted correctly as pNETs (sensitivity 71.4%).

Multiple studies have evaluated the possibility of predicting the histological grade of a pNET with CT-based texture analysis [24]. Some authors focused on the distinction between grade 1 and grade 2/3 pNETs, according to the different degrees of surgical resection needed (parenchyma-sparing vs. radical) and reported the performance of the tested nomogram (radiomics + clinical features) with an AUC of 0.894-0.902, which was higher than those of the single variables [25]. Moreover, Liang et al. [26] described a significant association between the Ki67 level and the rate of nuclear mitosis as an expression of cell proliferation, and a significant difference in overall survival between the G1 and G2/3 groups, predicted by the nomogram, as further confirmation of its prognostic value. As opposed to the previous studies, Guo et al. [27] tested radiomics features to distinguish pNETs with grade 1/2 or grade 3 with histopathological analysis, because the latter usually need chemotherapy or radiotherapy in addition to surgery. They found that, among conventional imaging features, arterial and portal enhancement ratios have the best sensitivity (86–94%) and specificity (92–100%), whereas among radiomics imaging features, mean gray-level intensity, entropy, and uniformity demonstrated good sensitivity (73–91%) and specificity (85–100%); when incorporating all these features together, the resulting AUC was ≥0.90.

##### Adenocarcinoma

For adenocarcinoma, radiomics and CT have been studied for differential diagnosis and survival prediction. Ren et al. [28] used CT and radiomics to assess their predictive ability in the differential diagnosis between pancreatic adenosquamous carcinoma (PASC) and PDAC. For this purpose, 81 patients with PDAC and 31 patients with PASC who underwent preoperative CECT were included. The authors selected seven radiomics features from late arterial phase images and three from the portal venous phase out of 792 radiomics features by using the random forest method extracted from the late arterial phase and portal venous phase. They validated their radiomics signature by using the 10-times leave-group-out cross-validation (LGOCV) method. Their signature showed promising results as a noninvasive method in the differential diagnosis between PASC and PDAC with 94.5% accuracy, 98.3% sensitivity, 90.1% specificity, 91.9% positive predictive value (PPV), and 97.8% negative predictive value (NPV).

Radiomics could be a supportive tool for the distinction of molecular phenotypes of PDAC that have different behaviors in terms of response to treatment and survival. A random forest (RF) ML algorithm by Kaissis et al. [29] was tested to distinguish between quasi-mesenchymal (QM − KRT81+) and non-quasi-mesenchymal (non-QM − HNF1a+) subtypes from radiomics features, reaching an AUC of 0.93, a sensitivity of 0.84, and a specificity of 0.92. Moreover, differences were reported in the median OS for identified QM and non-QM tumors at 16.1 and 20.9 months, respectively (HR 1.59).

Considering the poor outcome of PDAC, multiple studies have tested the application of radiomics in prognosis prediction, especially in patients that underwent curative surgery at risk for postoperative recurrence [30,31]. According to Xie et al. [32], a CT-based Rad-score with five features resulted in an independent prognostic factor for disease-free survival (DFS, HR 2.556) and overall survival (OS, HR 3.741) and patients with a higher Rad-score demonstrated a significantly poorer prognosis. No significant correlation was found between Rad-score and tumor recurrence. The combination of texture parameters with clinical data (differentiation grade, CA19-9, and TNM stage) into a radiomics nomogram could be a good survival estimator, also outperforming the clinical model and the TNM staging, with a C-index of 0.697 in the DFS analysis and 0.726 in the OS analysis.

Other authors focused on specific radiomics features, especially related to tumor heterogeneity and its prognostic role. Kim et al. [33] evaluated the differences in gray-level non-uniformity (GLN) as a texture parameter, and also created a Kaplan–Meier survival curve. After ROI placement on arterial CT images, significantly higher GLN values were found in tumors compared to normal pancreatic tissue and in cases with T3 diseases or poorly differentiated tumors. After multivariate analysis, a high GLN135 value, i.e., increased tissue heterogeneity, was statistically associated with a poor prognosis, and consequently, with short recurrence-free survival. Moreover, the presence of a non-uniform texture reflected an increased biological aggressiveness of the tumor, considering the nodal stage and the tumor differentiation. Authors further highlighted the prognostic role of texture analysis through the identification of histopathological features of the tumor, correlating the lower uniformity of pixel values and the higher level of hypoxia markers. Eilaghi et al. [34] described that uniformity, entropy, and correlation are significantly different in healthy and diseased parenchyma, but they are not associated with survival. On the contrary, the dissimilarity and inverse difference normalized were significantly related to OS, both with an AUC of 0.716, overcoming tumor intensity and tumor size.

The presence of nodal metastases is another indicator of poor prognosis after surgery that, according to Fang et al. [35], could be predicted with a texture analysis of a preoperative CT scan. In their cohort of 155 operable patients (73 with and 82 patients without nodal involvement), 10 texture features were significantly different in the two groups. The SumAverage, i.e., the measure of overall image brightness, resulted in being the most common co-occurrence matrix-based feature on portal venous images that indicated the overall density of the CT image, either at 1.25 mm or 5 mm. Differently, Li et al. [36] developed a combined prediction nomogram, based on the pathological grade, CT-based node status and a radiomics signature of 15 features that yielded an AUC of 0.912.

#### 3.1.2. Non-Oncological Applications

As for non-oncological application, there is still little evidence about CT radiomics and its implications with pancreatitis. While some authors have looked into the prediction of recurrence [37], others tried to evaluate whether a radiomics model could differentiate between acute and chronic pancreatitis [38].

##### Pancreatitis

Radiomics has been also tested in the support of non-oncological diagnosis, mainly in the identification of recurrent acute pancreatitis (AcP). Chen et al. [37] evaluated texture features on CT images at the first AcP episode to build a radiomics model of 10 features. This radiomics predictive model reached an AUC of 0.929, an accuracy of 89%, a sensitivity of 83.8%, and a specificity of 97.7%, outperforming the clinical model (an AUC of 0.671, an accuracy of 61%, a sensitivity of 60.5% and a specificity of 62.2%). Mashayekhi et al. [38] also included functional abdominal pain and chronic pancreatitis (CP) in the differential diagnosis with recurrent AcP. Their radiomics model included 11 features, 10 of which belonged to the gray-level co-occurrence matrix (GLCM) category and demonstrated high performance in the distinction of recurrent AP from nonspecific abdominal pain with AUC values of 0.77–0.95 and from CP with AUC values of 0.73–0.92. Then, an overall predictive accuracy of 82.1 % was found for the three diagnoses with an IsoSVM classifier, and recurrent AcP had the lowest rate of misclassification (5% vs. 21% for abdominal pain and 25% for CP).

Table 1 provides a summary of the papers included in the review, focused on the application of radiomics in CT images.

### 3.2. Radiomics and PET-CT

#### 3.2.1. Oncological Applications

Some PET-based radiomics studies have also been proposed [3,4,39,40,41,42,43,44,45,46]. PET images help in the characterization of biological features of tumors, which are usually associated with their sensitivity and/or aggressiveness [4]. Moreover, [^18^F]F-fluorodeoxyglucose (FDG) positron emission tomography/computed tomography (PET/CT) combines functional information and anatomic information [3].

Tumor delineation is one of the factors that may affect radiomics features. PET images are considered to be difficult to delineate as edge contours of uptake regions are not sharp and clear. Belli et al. [4] investigated the impact of delineation variability on PET radiomics features. For this purpose, they included 25 pancreatic cancer patients previously treated with FDG PET/CT. The authors found that PET_edge was sufficiently robust against manual delineation, which suggests the possibility of replacing manual with semiautomatic delineation of pancreatic tumors. In a different study, Zhang et al. [39] concluded that the quantified radiomics model is significantly superior to both human doctors and clinical factor-based prediction models in terms of accuracy and specificity for the differentiation of autoimmune pancreatitis and PDAC in F-FDG PET/CT images. According to their results, the combination of the SVM-RFE feature selection strategy and linear SVM classifier had the highest diagnostic performance, with an AUC of 0.93.

##### pNET

Other studies have assessed the potentiality of radiomics analysis extracted by [^68^Ga]Ga-DOTATOC PET/CT [40,41]. Mapelli et al. [40] found that specific texture features derived from preoperative [^68^Ga]Ga-DOTATOC and ^18^F-FDG PET/CT could noninvasively predict specific tumor characteristics and outcomes of patients with NETs. Meanwhile, Liberini et al. [41] carried out a pilot study on two NET patients. Their preliminary results suggested the use of RFs and TLSREwb-50 and SRETVwb-50 as parameters to evaluate the response to peptide receptor radionuclide therapy (PRRT) in their patients.

##### Adenocarcinoma

Lim et al. [42] investigated FDG PET/CT images of 48 patients with PDAC. Their results showed that genetic alterations of KRAS (correlated with reduced low-intensity textural signatures) and SMAD4 (correlated with reduced high-intensity textural signatures) had significant associations with FDG PET-based radiomics features in PDAC.

Moreover, Xing et al. [3] developed and validated a model based on radiomics features derived from [^18^F]F-fluorodeoxyglucose PET/CT images to preoperatively predict the pathological grade of PDACs. Their model, based on a twelve-feature-combined radiomics signature, could stratify PDAC patients into grade 1 and grade 2/3 groups with an AUC of 0.994 in the training set and 0.921 in the validation set.

Other studies focused on the identification of prognostic features in FDG PET/CT images in patients treated with stereotactic body radiation therapy. Cui et al. [43] included 139 patients with locally advanced pancreatic cancer that underwent the PET/CT study for the planning of the radiation treatment, and they identified a prognostic signature of seven features, including texture feature, shape complexity, and SUV intensity distribution. Through multivariate analysis, the proposed model was significantly associated with overall survival with a hazard ratio of 3.72 and outperformed conventional imaging parameters, i.e., gross tumor volume and maximum standardized uptake value (SUVmax). Yue et al. [44] identified five significant prognostic variables with multivariate Cox analysis (age, node stage, variations of homogeneity, variance, and cluster tendency) that are able to predict therapy response using both pre- and post-treatment FDG PET/CT. Specifically, they evaluated the texture variation between the two examinations as an index of locoregional metabolic response: a lower texture variation (<15%) was found in the high-risk group with a shorter mean overall survival (17.7 months).

Liu et al. [45] developed a radiomics-based prediction model using dual-time PET/CT imaging for the noninvasive classification of PDAC and autoimmune pancreatitis (AIP) lesions. In their series, they included 112 patients (48 patients with AIP and 64 patients with PDAC). Their model was developed from a combination of the SVM-RFE and linear SVM with the required quantitative features. The multimodal and multidimensional features obtained an average AUC of 0.9668, an accuracy of 89.91%, a sensitivity of 85.31%, and a specificity of 96.04%.

Toyama et al. [46] evaluated the prognostic value of FDG PET radiomics and found that the gray-level zone length matrix (GLZLM) gray-level non-uniformity (GLNU) PET parameter was the most relevant factor for predicting 1-year survival, followed by total lesion glycolysis (TLG). The combination of GLZLM, GLNU, and TLG stratified patients into three groups according to the risk of poor prognosis.

Table 2 provides a summary of the papers included in the review, focused on the application of radiomics in PET-CT images.

### 3.3. Radiomics and MRI

#### 3.3.1. Oncological Applications

MRIs offer several advantages in the diagnosis of pancreatic tumors, such as contrast resolution and a better examination of the pancreaticobiliary system [47].

When evaluating cystic lesions with MRI, one of the daily challenges of a radiologist is examining IPMNs and expressing the likelihood of malignant degeneration. On this note, some authors investigated the use of MR radiomics signatures to predict the risk of malignancy of IPMNs through texture analysis alone [48] or combining both clinical characteristics and radiological characteristics [49].

As previously stated, the radiomics of pNETs has been studied thoroughly for their immediate clinical implications. When it comes to MRI findings, some authors [50] have investigated radiomics models that could predict the histopathologic grade of pNETs. On the other hand, other authors have focused mainly on differentiating pNETs from SPNs, either with T1WI and postcontrast phases [51] or with the use of DWI sequences together with other standard phases [52].

As for adenocarcinoma, most studies about the radiomics of MRI rely on ADC metrics either to differentiate normal parenchyma from pancreatic neoplasm [53] or for outcome prediction and overall survival [54].

##### Cystic Lesions

Jeon et al. [48] studied the utility of MR findings and texture analysis for predicting the malignant potential of pancreatic IPMNs. The authors found seven significant predictors of malignancy: effective diameter, surface area, sphericity, compactness, entropy, and gray-level co-occurrence matrix entropy (*p*  <  0.05). They calculated the diagnostic performance by using Cohen’s κ for predicting malignant IPMNs, which was 0.80 (good agreement). Recently, Cui et al. [49] assessed the use of a nomogram combining clinical characteristics and radiomics features, including histograms, texture parameters, the RLM (run-length matrix), and the GLCM (gray-level co-occurrence matrix), for the diagnosis of high-grade branching-type IPMNs in 202 patients from three medical centers. Their radiomics signature obtained AUC values of 0.836 in the training cohort, 0.811 in the first external validation cohort, and 0.822 in the second external validation cohort, whereas using the radiomics nomogram, the high-grade disease-associated AUC values were 0.903 (training cohort), 0.884 (external validation cohort 1), and 0.876 (external validation cohort 2). Their radiomics nomogram model could effectively distinguish high-grade patients with IPMN preoperatively, resulting in better treatment methods and tailored therapy in patients with IPMN.

##### pNET

Regarding pNETs, Guo et al. [50] proved that MRI findings, including tumor margin, texture, local invasion or metastases, tumor enhancement, and diffusion restriction, as well as texture parameters, can aid in the prediction of pNET grading. For this purpose, they evaluated the performance of MRI findings and texture parameters for the prediction of the histopathologic grade of a pNET with 3-T magnetic resonance. They included 31 G1, 29 G2, and 17 G3 patients. G2/G3 tumors showed higher frequencies of an ill-defined margin, a predominantly solid tumor type, local invasion or metastases, hypoenhancement in the arterial phase, and restriction diffusion. The AUCs of six predicting models on T2WI and DWI ranged from 0.703 to 0.989. Song et al. [51] assessed the value of radiomics parameters derived from MRI in the differentiation of hypovascular nonfunctional pancreatic neuroendocrine tumors (hypo-NF-pNETs) and solid pseudopapillary neoplasms of the pancreas (SPNs) by including fifty-seven SPN patients and twenty-two hypo-NF-pNET patients. They extracted radiomics features from the T1WI, arterial, portal, and delayed phases of MR images. The radiomics signature of the arterial phase was picked to build a clinic-radiomics nomogram. The nomogram, composed of the age and radiomics signature of the arterial phase, showed sufficient performance for discriminating SPNs and hypo-NF-pNETs with AUC values of 0.965 and 0.920 in the training and validation cohorts, respectively. Similarly, Li et al. [52] tested preoperative MRI-based texture analysis to differentiate NF-pNETs (201 patients) and SPNs (101 patients). Nonlinear discriminant analysis (NDA) had a lower value of misclassification rate, especially with DWI sequences (7.92%) than radiologists (34.65%), and the postcontrast DCE-T1WI+fs sequence appeared to provide more information for the distinction with mean and percentile as the most discriminative features. 

##### Adenocarcinoma

In adenocarcinoma, MR and radiomics have shown great results in differentiating normal pancreatic parenchyma from pancreatic neoplasm. In 2019, Taffel et al. [53] evaluated whole-lesion 3D histogram apparent diffusion coefficient (ADC) metrics for the assessment of pancreatic malignancy. For this purpose, forty-two patients with pancreatic malignancies (36 PDACs, 6 PanNETs) had undergone abdominal MRI with diffusion-weighted imaging before endoscopic ultrasound biopsy or surgical resection. The volumetric ADC histogram metrics showed to be effective as a noninvasive biomarker of pancreatic malignancy with an AUC = 0.787–0.792.

The identification of the specific PDAC subtypes could have an important prognostic role in a different rate of treatment response and changes in the overall survival. PDAC has a high heterogeneity on a genetic, proteomic, and transcriptomic level that cannot be appreciated on conventional imaging techniques. Kaissis et al. [54] developed an ML model for the extraction of radiomics features from the diffusion-weighted imaging (DWI)-derived ADC maps for a prognostic evaluation of the tumor. Their algorithm achieved high performances in the outcome prediction with 87% sensitivity, 80% specificity, and 90% AUC in the distinction of above- versus below-median overall survival and the main indicative imaging features belonged to the heterogeneity-related group. Moreover, according to the histopathological classification, almost all the patients with a quasi-mesenchymal tumor subtype (8/9) demonstrated a below-median overall survival. The same research group performed a similar study, focusing on molecular PDAC subtypes and response to chemotherapy. Specifically, KRT81+ patients had a significantly lower median overall survival than KRT81- patients (7.0 vs. 22.6 months, HR 4.03) and a better response to gemcitabine-based chemotherapy over FOLFIRINOX (10.14 vs. 3.8 months median overall survival, HR 2.33). On the contrary, FOLFIRINOX was more effective in KRT81- patients than the gemcitabine-based treatment (30.8 vs. 13.4 months median overall survival, HR 2.41) [55].

#### 3.3.2. Non-Oncological Applications

In 2016, Becker et al. [56] demonstrated that b-values significantly affect texture analysis on DWI images. To this purpose, echo-planar DWI sequences at 16 b-values ranging between 0 and 1000 s/mm2 were acquired at 3-T in 8 healthy male volunteers. According to the authors, several texture features vary systematically in healthy tissues at different b-values, which needs to be taken into account if DWI data with different b-values are analyzed.

##### Pancreatitis

Radiomics models have been applied to the study of pancreatitis whose diagnosis is mainly based on morphological changes in the pancreas using conventional imaging techniques [57,58]. In the very early phases of acute pancreatitis, those pancreatic abnormalities are not easy to evaluate and could lead to underestimating the incidence of the disease. Starting from this idea, Lin et al. [57] developed a radiomics model using contrast-enhanced MRI (CE-MRI), specifically on the portal venous phase images, for the prediction of the AcP severity also in the early stages of the disease. Their model had an AUC of 0.848 in the training cohort and outperformed the conventional scoring systems, i.e., the MR severity index (MRSI; AUC 0.719), Acute Physiology and Chronic Health Evaluation (APACHE II; 0.725), and bedside index for severity in AcP (BISAP; AUC 0.708).

In 2020, Frokjaer et al. [58] assessed the texture analysis in MRI examinations from 77 patients with CP and 22 controls and obtained a 97% sensitivity, 100% specificity, and 98% accuracy in the classification of chronic pancreatitis vs. healthy controls.

Table 3 provides a summary of the papers included in the review, focused on the application of radiomics in MRI images.

### 3.4. Radiomics and PET-MRI

#### Oncological Applications

A study by Gao et al. [59] assessed the use of PET-MRI and radiomics for oncologic treatment prediction outcomes. Specifically, they focused on the imaging biomarkers of glucose metabolic activity and DWI derived from pretreatment integrated ^18^F-fluorodeoxyglucose PET-MRI imaging as potential predictive factors of metastasis in patients with PDAC. The AUC was 0.939, 0.894, 0.924, and 0.909 for PET-GLRLM_LRHGE, ADC-GLRLM_LRHGE, ADCGLRLM_GLNU, and ADC-GLRLM_RLNU, respectively, whereas the logistic regression model with proposed features obtained an AUC of 1.000.

Table 4 provides a summary of the papers included in the review, focused on the application of radiomics in PET-MRI images.

### 3.5. Radiomics in Combined CT and MRI Studies

#### Oncological Applications

Other studies included radiomics applied to both CT and MRI. In 2019, Azoulay et al. [60] compared morphological imaging features and CT texture histogram parameters between grade 3 pancreatic neuroendocrine tumors (G3-pNETs) and neuroendocrine carcinomas (NECs). For this purpose, they included patients with pathologically proven G3-pNETs and NECs who had CT and MRI examinations between 2006–2017 and were retrospectively included. Two radiologists reviewed both CT and MRI for tumor size, enhancement patterns, hemorrhagic content, liver metastases, and lymphadenopathies, and a texture histogram analysis of tumors was performed on arterial and portal phase CT images. The authors found that pancreatic NECs are larger, more frequently hypoattenuating, and more heterogeneous with hemorrhagic content than G3-pNETs on CT and MRI with an AUC of 0.694, 78% sensitivity, and 58% specificity. Recently, Ohki et al. [61] used ADC values in the differential diagnosis of malignant pancreatic disease. They found that texture analysis may aid in differentiating between G1 and G2–3-pNETs.

Table 5 provides a summary of the papers included in the review, focused on the application of radiomics in CT and MRI images.

## 4. Insights on CAD Applied to Pancreatic Imaging

**Figure 3:**
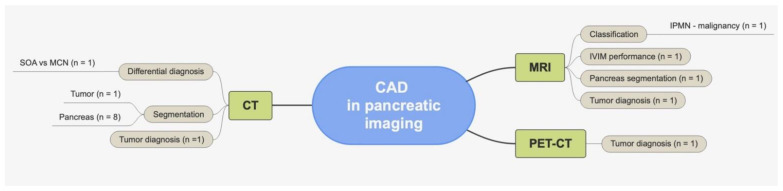
Summary of CAD applications in pancreatic imaging.

In the last few years, multiple CAD software approaches have been developed and tested on pancreatic imaging to improve the accuracy of examinations and the clinical decision-making process [62,63,64,65,66,67,68,69,70,71,72,73,74,75,76,77]. They have shown promising results for segmentation [65,66,67,68,69,70,71,72,77], tumor diagnosis, and classification [63,64,73,74,75] (Figure 3).

### 4.1. CAD and CT

The use of CT is routine for the diagnosis and follow-up of patients with pancreatic cancer. The use of CAD can help doctors improve diagnostic efficiency and accuracy [63,64,65], which does not depend directly on the subjective judgment and experience of the single physician. Even so, the application of CAD in pancreatic CT imaging may be difficult due to a lack of contrast between pancreatic parenchyma and bowel, large variations in the size of the pancreatic volume, and large variations in peripancreatic fat tissue [62].

#### 4.1.1. Oncological Applications

Liu et al. [63] aimed to diagnose pancreatic cancer using a convolutional neural network (CNN) classifier for CECT images. For this purpose, they used three different datasets. The first dataset had 295 patients with pancreatic cancer and 256 controls for training, and 75 patients with pancreatic cancer and 64 controls for validation. The second dataset consisted of 101 patients with pancreatic cancer and 88 controls, whereas the third dataset had 281 pancreatic cancer subjects and 82 controls. In all three sets, their model obtained an accuracy of more than 80%. The authors compared the use of the CNN in distinguishing pancreatic cancer from noncancerous tissue in CT to radiologist interpretation. Their CNN-based analysis achieved higher sensitivity than radiologists did (0.983 vs. 0.929, difference 0.054, *p* = 0.014). The CNN missed 3 (1.7%) of 176 pancreatic cancers (1.1–1.2 cm). Radiologists missed 12 (7%) of 168 pancreatic cancers (1.0–3.3 cm), of which 11 (92%) were correctly classified using the CNN. Finally, their CNN model obtained a sensibility of 92.1% for tumors smaller than 2 cm.

##### Cystic Lesions

Li et al. [64] assessed the effectiveness of a CAD scheme including tumor size, contour, location, and low-energy CT values in the differential diagnosis of pancreatic serous oligocystic adenomas (SOAs) from MCNs of PCNs using conventional and additional quantitative spectral CT features. The authors concluded that by combining conventional features with additional spectral CT features, the CAD scheme improved the overall accuracy from 88.37% to 93.02%. Reena Roy et al. [65] used a model for both an automated whole pancreas and PCN segmentation of CT images in oncologic patients using inter-/intraslice circumstantial instruction with preprocessing, segmentation, feature extraction, and classification. The authors found that the combination of various algorithms such as K-means, feature extraction using GLCM, and segmentation and classification using an artificial neural network (ANN) provides better results with increased efficiency, resulting in better classification of the pancreatic cysts.

#### 4.1.2. Non-Oncological Studies

The main application of DL algorithms for non-oncological studies was the automatic segmentation of pancreas and pancreatic lesions, which can support diagnosis and treatment planning and reduce the workload. [66,67,68,69,70,71,72] Gibson et al. [66] used a deep learning-based segmentation algorithm for eight organs including the pancreas. Their model achieved a Dice similarity coefficient (DSC) of 0.78 vs. 0.71, 0.74, and 0.74. Similarly, Xue et al. [67] also worked on automated pancreas segmentation by using a cascaded multitask 3D fully convolutional network (FCN). Their method achieved a Dice score of 86.9. Zheng et al. [68] designed a 2.5D CNN in an automatic pancreas segmentation framework using 3D CT scans. Their method obtained a Dice similarity coefficient, sensitivity, and specificity of 86.21%, 87.49%, and 85.11%, respectively. However, some of the problems of automated DL segmentation performance in pancreas CT are poor gray-value contrast and the complex anatomy of the pancreas. To correct this situation, Boers et al. [62] developed a UNet they called iUnet to improve the quality of the colors. The performance for manual segmentation by a radiologist was 87% in 15 min, whereas the semiautomatic (radiologist + UNet) segmentation performance was 86% in 8 min.

Suman et al. [69] used the CTNVIDIA 3D slicer segmentation module, a DL model, for obtaining pancreatic segmentation. They used CECT scans that were previously reported as negative or unremarkable in the pancreatic region and obtained a DSC of 63%. Similarly, Nishio et al. [70] used CT images and a combination of DL and data augmentation to automatically segment the pancreas. The data augmentation method used included a mix-up, and random image cropping and patching (RICAP). Four-fold cross-validation was performed to train and evaluate these models with data augmentation methods and obtained a DSC of 0.703–0.789.

The interclass indistinction problem occurs as the density of the surrounding tissue is similar to that of the pancreas, resulting in the surrounding tissue being grouped with the pancreas, whereas the intraclass inconsistency occurs when the middle part of the pancreas is mistaken for the background, resulting in incomplete pancreas segmentation. Recently, Li et al. [71] focused on solving the issues of intraclass inconsistency and interclass indistinction in pancreas segmentation. To do this, they improved the contextual and semantic feature information acquisition method of the biomedical image segmentation model (UNet) based on a convolutional network and proposed an improved segmentation model called the multiscale attention dense residual U-shaped network (MAD-UNet). By using this new approach, the authors were able to reduce the effects of intraclass inconsistency and obtained a DSC of 86.10%.

Panda et al. [72] developed two-stage 3D CNNs for fully automated volumetric segmentation of the pancreas on CT. They also evaluated its performance in the context of intra-reader and inter-reader reliability at 1917 abdomen full-dose and reduced-radiation-dose CTs on a public dataset. Their 3D CNN obtained a DSC of 0.91 (0.03); they also obtained good reliability between model and R1 in both full- and reduced-dose CT (full-dose DSC: 0.81 (0.07), CCC: 0.83; reduced-dose DSC: 0.81 (0.08)).

Table 6 provides a summary of the papers included in the review, focused on the application of CAD in CT images.

### 4.2. CAD and PET-CT

Li et al. [73] extracted major structure and location information from the ROI on CT and PET images using a shape model-based algorithm. The algorithm used a collection of pancreas-shaped models. Li et al. achieved a 96.47% accuracy for PDAC classification in 80 cases with a 95.23% sensitivity and 97.51% specificity.

Table 7 provides a summary of the papers included in the review, focused on the application of CAD in PET-CT images.

### 4.3. CAD and MRI

The use of MRI as a soft-tissue contrast and noninvasive method is of great importance in the medical field, and even more now thanks to the use of CAD applied to oncological imaging [74,75].

#### 4.3.1. Oncological Applications

Balasubramanian et al. [74] combined the response of ANN and support vector machine (SVM) techniques for pancreatic tumor classification. They used GLCM for extracting features from MR images of the pancreas and selected the best features using the JAFER algorithm. These features then were analyzed by five classification techniques: ANN BP, ANN RBF, SVM Linear, SVM Poly, and SVM RBF. Their results showed that the ANN BP technique has a 98% classification accuracy.

##### Cystic Lesions

In a more recent study, Donofrio et al. [75] evaluated the diagnostic accuracy of dynamic MRI with DWI sequences in the identification of mural nodules of pancreatic IPMN by using pathological analysis as the gold standard. They performed a histogram analysis of the distribution of ADC and their results showed that entropy corresponded to the best J Youden index of 0.48 with a sensitivity of 68.75%, and a specificity of 79.25% in the distinction between a lesion with low-grade dysplasia and one with high-grade dysplasia. They also found that MRI with dynamic and DWI sequences was an accurate method for the identification of 5mm solid nodules of the IPMN, which correlated with the lesion malignancy.

#### 4.3.2. Non-Oncological Applications

Barbieri et al. [76] prospectively assessed the feasibility of training a DNN for an intravoxel incoherent motion (IVIM) model fitting to diffusion-weighted MRI (DW-MRI) data. Two independent readers delineated regions of interest in the pancreas. DNNs were trained for IVIM model fitting using these data; results were compared to least-squares and Bayesian approaches to IVIM fitting. Their approach had high consistency between two readers (ICCs between 50% and 97%).

Chen et al. [77] developed a DL technique for fully automated pancreas segmentation. Their model took in multislice MR images and generated the output of the segmentation results, obtaining a DSC of 0.88. Their DL-based technique named ALAMO was found useful for fully automated multiorgan segmentation on abdominal MRI.

Table 8 provides a summary of the papers included in the review, focused on the application of CAD in MRI images.

## 5. Discussion

For pancreatic pathologies, the use of medical imaging is essential for diagnosis, evaluation, and treatment planning [78]. The application of AI and radiomics is emerging and expanding, especially with regard to their applications for non-oncological pancreatic segmentation and tumor differentiation. In this review, we divided articles depending on their focus on CAD or radiomics, and further divided into two macro-categories based on their topic: non-oncological and oncological studies.

As concerns the non-oncological studies included in this review, the main application is DL segmentation, which is a useful step for training AI algorithms for detecting, characterizing, and classifying pancreatic lesions. As for the oncological studies included, AI is mainly used both for differential diagnosis and lesion segmentation. PDAC, which is the most prevalent neoplastic disease of the pancreas, has been extensively studied in the literature along with pNETs, especially in those studies focused on differential diagnosis.

Although great developments have occurred in recent years, it is important to address the obstacles that still need to be overcome before these technologies can be implemented into our clinical routines. However, despite the surge in publications on pancreas CAD and radiomics, there has not been a clinical translation of these applications.

Despite the use of large sample sizes in some studies and a large number of extracted features in radiomics, the limited heterogeneity of labeled training datasets may be one of the reasons that have precluded the clinical-grade performance and generalizability of the CAD and radiomics models. The articles reviewed were mainly retrospective with insufficient clinical data; for this reason, it is necessary to carry out more prospective studies that combine AI/radiomics and clinical data. Moreover, most of the articles included considered only the venous phase in CT for assessing pancreatic lesions, whereas only some of them included both arterial and venous phases in the study of lesion characteristics and showed better results than those who considered just the venous phase.

According to Chen et al. [78], there are three main challenges to the application of AI in pancreatic imaging. The first one is the inconsistencies and contradictions found in the results of the studies. For this reason, the authors proposed that there should be an initiative to standardize the development of quantitative imaging biomarkers. Second, there is a need for having more public annotated data on pancreatic imaging as the available data are not enough; this is due to the labor-intensive work that needs to be performed by experienced radiologists to label target lesions. Furthermore, the majority of the available studies are retrospective, with limited clinical, laboratory, and outcome data that prevent AI from being applied in clinical practice.

We are aware that the narrative nature of our review represents a limitation due to the absence of a systematic approach during the study selection process. We designed our study to provide only an overview of the available literature on the application of CAD/radiomics in pancreatic imaging.

## 6. Conclusions

This review demonstrated that AI applied to pancreatic imaging represents promising tools for a noninvasive diagnosis that will allow personalized approaches to patients. Up to now, the use of CAD and radiomics in pancreatic imaging has proven to be useful for both non-oncological and oncological purposes. There is much excitement and optimism about their applications; however, more multicenter, prospective, and large-scale studies need to be performed to introduce these tools into clinical practice.

## Figures and Tables

**Table 1:** Applications of radiomics in pancreatic CT images.

Author	Year	Radiomics Analysis	Task	N Pts	Data Split	Ref Standard	CT Phase	Results
Yang	2019	LIFEx software	Differential diagnosis (MCN vs. SCN)	78 (25 MCNs, 53 SCNs)	RW (TS:DS = 4:1)	Histopathology	AP, PVP	Radiomics features, 2 mm: AUC 0.66, Acc 74%, Sen 86%, Spe 71% Radiomics features, 5 mm: AUC 0.75, Acc 83%, Sen 85%, Spe 83%
Yang (1)	2019	LIFEx software	Differential diagnosis (MCN vs. SCN)	91 (32 MCNs, 59 SCNs)	SW	Histopathology	PAP	Textural features: AUC 0.777 Textural features + morphological characteristics: AUC 0.893
Xie	2019	In-house algorithm (MATLAB R2017a)	Differential diagnosis (MCN vs. SCN)	57 (31 MCNs, 26 SCNs)	SW	Radiologist	AP, PVP, DP	Radiomics model: AUC 0.989, Acc 94.7%, Sen 93.6%, Spe 96.2% Combined model (radiomics + radiological features): AUC 0.994, Acc 98.2%, Sen 96.8%, Spe 100%
Chen	2021	Analysis Kit Software (v 3.0.0.R)	Differential diagnosis (PCN vs. SCN)	89 (31 SCNs, 30 IPMNs, 28 MCNs)	RW (63 TS, 26 VS)	Radiologist	NECT, AP, PVP	Radiomics signature NECT + AP + PVP: AUC 0.817
Wei	2019	NS	Differential diagnosis (PCN vs. SCN)	260 (102 SCNs, 158 non-SCNs)	SW (200 TS, 60 VS)	Radiologist	AP, PVP	Radiomics method: AUC 0.837, Sen 66.7%, Spe 81.8%
Shen	2020	ANN, RF, SVM (MATLAB 2017b)	Differential diagnosis (PCN)	164 (76 SCAs, 40 MCNs, 48 IPMNs)	SW (115 TS, 41 VS)	Histopathology	AP	Radiomics model (nine features) Acc 71.43% (SVM, ANN), 79.59% (RF)
He	2019	Pyradiomics	Differential diagnosis (PDAC vs. pNET)	147 (80 PDACs, 67 pNETs)	SW (100 TS, 47 VS)	Radiologist	PAP, PVP	Radiomics signature: AUC 0.873, Acc 76.6%, Sen 92.3%, Spe 70.6% Integrated model (radiomics + clinical features): AUC 0.884, Acc 80.4%, Sen 80.0%, Spe 80.8%
Li	2018	FireVoxel Software	Differential diagnosis (PDAC vs. pNET)	75 (50 PDACs, 25 pNETs)	SW	Radiologist	AP, PVP	Combined fifth + skewness as the best parameters: AUC 0.887, Sen 90%, Spe 80%
Reinert	2020	Pyradiomics	Differential diagnosis (PDAC vs. pNET)	95 (53 PDACs, 42 pNETs)	SW	Radiologist	PVP	Significant discriminatory features: first-order features, i.e., median, total energy, energy, 10th percentile, 90th percentile, minimum, maximum; second-order feature, i.e., gray-level co-occurrence matrix informational measure of correlation (Sen 79%, Spe 71%)
Yu	2020	Analysis Kit Software	Differential diagnosis (PDAC vs. pNET)	120 (80 PDACs, 40 pNETs)	RW	Radiologist	AP, PVP	AP texture model: AUC 0.855 PVP texture model: AUC 0.929
Ren	2020	Analysis Kit Software (v 3.0.0.R)	Differential diagnosis (PDAC vs. PASC)	112 (81 PDACs, 31 PASCs)	RW (TS:DS = 2:1)	Histopathology	PAP, PVP	Acc 94.5%, Sen 98.3%, Spe 90.1%, PPV 91.9%, NPV 97.8%
Tobaly	2020	Pyradiomics (v 2.2.0)	IPMN grading	408 (181 benign, 227 malignant)	SW (296 TS, 112 VS)	Histopathology	PAP, PVP	Benign vs. malignant IPMN radiomics model: AUC 0.71, Acc 64%, Sen 69%, Spe 57% Radiomics + surgical indication: AUC 0.75, Acc 67%, Sen 69%, Spe 65%
Hanania	2016	IBEX	Prediction of IPMN malignancy	53 (34 high-grade, 19 low-grade)	SW(TS:DS = 7:3)	Histopathology	AP	Radiomics panel (10 features): AUC 0.96, Sen 97%, Spe 88%
Permuth	2016	In-house algorithm (Definiens Platform)	Prediction of IPMN malignancy	38 (20 benign, 18 malignant)	SW(TS:DS = 9:1)	Histopathology	AP, PVP	Radiomics signature (14 features): AUC 0.77, Sen 83%, Spe 74% Integrated model 1 (radiomics + genomic data): AUC 0.92, Sen 83%, Spe 89% Integrated model 2 (radiomics + standard imaging + genomic data): AUC 0.93, Sen 89%, Spe 89%
Canellas	2018	TexRAD (v 3.1)	pNET grading	101 (63 grade 1, 35 grade 2, 3 grade 3)	SW	Histopathology	PVP	Entropy as an independent predictor: OR 3.7, AUC 0.65, values > 4.65 with differences in DFS (G1 vs. G2/G3)
Gu	2019	Pyradiomics (v 1.3.0)	pNET grading (G1 vs. G2/G3)	138 (57 grade 1, 69 grade 2, 12 grade 3)	RW (104 TS, 34 VS)	Histopathology	AP, PVP	Nomogram (radiomics features + clinical risk factor tumor margin): AUC 0.902
Guo	2019	MATLAB R2014a	pNET grading (G1/G2 vs. G3)	37 (13 grade 1, 11 grade 2, 13 grade 3)	RW	Histopathology	NECT, AP, PVP	Texture features AUC 0.93, Sen 91.7%, Spe 84.6% Size/margin + texture features AUC 0.958, Sen 91.6%, Spe 87.5%
Liang	2019	In-house algorithm (MATLAB R2016a)	pNET grading (G1 vs. G2/G3)	137 (70 grade 1, 67 grade 2/3)	RW (86 TS, 51 VS)	Histopathology	AP	Nomogram (eight radiomics features + clinical stage): AUC 0.891
D’Onofrio	2019	MaZda Software (v 4.6)	pNET grading	100 (31 grade 1, 52 grade 2, 17 grade 3)	RW	Radiologist	AP, PVP	Kurtosis is different among three G groups: AUC 0.924, Sen 82%, Spe 85% for G3 diagnosis Entropy different between G1 and G3 and G2 and G3 groups: AUC 0.732, Sen 82%, Spe 64% for G3 diagnosis
Kaissis	2020	Pyradiomics	PDAC classification	207 (45 QM, 136 non-QM, 26 unclassifiable)	SW (181 TS, 26 VS)	Histopathology	PVP	AUC 0.93, Sen 0.84, Spe 0.92
Attiyeh	2018	MATLAB R2015a	PDAC prognosis	161	SW (113 TS, 48 VS)	Radiologist	PVP	Model A, preoperative CA19-9 and image features: c-index 0.69 Model B, preoperative CA19-9, Brennan score (postresection pathological variables), and image features: c-index 0.74
Khalvati	2019	Pyradiomics	PDAC prognosis	98	SW (30 TS, 68 VS)	Radiologist	PAP, PVP	Radiomics signature: HR 1.35 (Reader 2), 1.56 (Reader 1)
Yun	2018	NS	PDAC prognosis	88 (70 recurrence, 18 non-recurrence)	SW	Radiologist	PAP, PVP	Correlation of recurrence with texture features Average: AUC 0.736, standard deviation: AUC 0.709, contrast: AUC 0.692, correlation: AUC 0.698 Survival analysis nodal metastasis: HR 2.0375, average: HR 0.5599, standard deviation HR 0.5745
Xie	2020	NS	PDAC prognosis	220	SW (147 TS, 73 VS)	NS	PAP	Rad-score: low-RS correlated with better prognosis (AUC 0.715), HR 2.556 for DFS, HR 3.741 for OS
Kim	2019	NS	PDAC prognosis	116	SW	Radiologist	AP	GLN135: higher levels correlated with shorter DFS (HR 6.030)
Eilaghi	2017	MATLAB R2015a	PDAC prognosis	30	SW	Radiologist	PAP, PVP	Prediction of OS Tumor dissimilarity: AUC 0.716 Inverse difference normalized: AUC 0.716
Fang	2020	MaZda Software (v 4.6)	Prediction of LN metastasis	155 (73 nodal matastases, 82 without nodal metastases)	RW	Histopathology	AP, PVP	Ten texture features with significance in ROC analysis: biggest AUC 0.630 for wavelet-based feature WavEnLH_s-2
Li	2020	Pyradiomics	Prediction of LN metastasis	159 (59 nodal matastases, 100 without nodal metastases)	SW (118 TS, 41 VS)	Histopathology	AP, PVP	Radiomics signature (15 features): AUC 0.912
Chen	2019	IBEX	AcP prognosis	389 (181 recurrent AcP)	RW (271 TS, 118 VS)	Radiologist	AP, PVP	Recurrence prediction: AUC 0.929, Acc 89.0%
Mashayekhi	2020	In-house algorithm (MATLAB)	Differential diagnosis (recurrent AcP vs. CP)	56 (20 recurrent AcP, 19 functional abdominal pain, 17 CP)	SW	Radiologist	PVP	Acc 82.1%; recurrent AP: AUC 0.88, Sen 95%, Spe 78%; CP: AUC 0.90, Sen 71%, Spe 95%

Acc—accuracy, ANN—artificial neural network, AP—arterial phase, AcP—acute pancreatitis, AUC—area under the curve, CECT—contrast-enhanced computed tomography, CNN—convolutional neural network, CP—chronic pancreatitis, CT—computed tomography, DFS—disease-free survival, DP—delayed phase, HR—hazard ratio, IPMN—intraductal papillary mucinous neoplasm, MCN—mucinous cystic neoplasm, NECT—non-enhanced computed tomography, NS—not specified, OR—odds ratio, OS—overall Survival, PAP—pancreatic phase, PASC—pancreatic adenosquamous carcinoma, PDAC—pancreatic ductal adenocarcinoma, PCN—pancreatic cystic neoplasm, pNET—pancreatic neuroendocrine tumor, PVP—portal venous phase, RF—random forest, RW—record-wise, SCA—serous cystic adenoma, SCN—serous cystic neoplasm, SVM—support vector machine, Sen—sensitivity, Spe—specificity, SW—subject-wise, TS—training set, VS—validation set.

**Table 2:** Applications of radiomics in pancreatic PET-CT images.

Author	Year	Radiomics Analysis	Task	N Pts	Data Split	Reference Standard	Radiotracer	CT Phase	Results
Liu	2021	SVM (MATLAB R2018a)	Differential diagnosis (PDAC vs. autoimmune pancreatitis)	112 (64 PDACs, 48 autoimmune pancreatitis)	RW	Radiologist	FDG	NECT	AUC 0.9668, Acc 89.91%, Sen 85.31%, Spe 96.04%
Zhang	2019	SVM (MATLAB R2017a)	Differential diagnosis (PDAC vs. autoimmune pancreatitis)	111 (66 PDACs, 45 autoimmune pancreatitis)	RW	Radiologist	FDG	NECT	AUC 0.93, Acc 85%, Sen 86%, Spe 84%
Lim	2020	MIM (v 6.4)	PDAC classification	48	SW	Radiologist	FDG	NECT	KRAS gene mutation: significant association with long-run emphasis (AUC 0.806), zone emphasis (AUC 0.794), large-zone emphasis (AUC 0.829); SMAD4 gene mutation: significant association with standardized uptake value skewness (AUC 0.727), long-run emphasis (AUC 0.692), high-intensity textural features such as run emphasis (AUC 0.775), short-run emphasis (AUC 0.736), zone emphasis (AUC 0.750), and short-zone emphasis (AUC 0.725)
Xing	2021	Pyradiomics	PDAC grading	149	RW (99 TS, 50 VS)	Nuclear medicine physician	FDG	NECT	Prediction model (12 features): AUC 0.921 for G1 vs. G2/3
Mapelli	2020	Chang-Gung Image Texture Analysis software package (v 1.3)	pNET prognosis	61	RW	NS	DOTADOC, FDG	NECT	DOTATOC PET: SZV, entropy, intensity variability, and SRD were predictive of tumor dimension; FDG PET: intensity variability, SZV, homogeneity, SUVmax, and MTV were predictive for tumor dimension
Liberini	2020	LIFEx software (v 5.10)	pNET prognosis	2	SW	NS	DOTADOC	NECT	A significant difference of 28 radiomics features in pre- and post-treatment studies
Toyama	2020	LIFEx software	PDAC prognosis	161	SW	Histopathology	FDG	NECT	GLZLM GLNU as an independent predictor factor for poor prognosis (HR 2.0)
Cui	2016	MITK software (v 3.1.0.A)	PDAC prognosis	139	SW (90 TS, 49 VS)	NS	FDG	NECT	Prognostic signature (seven features): HR 3.72
Yue	2017	3D kernel-based approach	PDAC prognosis	26	SW	NS	FDG	NECT	Low-risk group: higher texture variation (>30%) and longer mean OS (29.3 months); high-risk group: lower texture variation (<15%) and shorter mean OS (17.7 months)
Belli	2018	CGITA software (v 1.4)	Tumor segmentation	25	SW	Radiologist	FDG	NECT	DSC 0.73

Acc—accuracy, AUC—area under the curve, CT—computed tomography, DOTADOC—DOTA-Tyr-octreotide, FDG—fluorodeoxyglucose, HR—hazard ratio, NECT—non-enhanced computed tomography, NS—not specified, OS—overall survival, PET—positron emission tomography, pNET—pancreatic neuroendocrine tumor, PDAC—pancreatic ductal adenocarcinoma, RW—record-wise, Sen—sensitivity, Spe—specificity, SW—subject-wise, TS—training set, VS—validation set.

**Table 3:** Applications of radiomics in pancreatic MRI images.

Author	Year	Radiomics Analysis	Task	N Pts	Data Split	Reference Standard	MRI Phase	Results
Song	2021	Pyradiomics	Differential diagnosis (NF-pNET vs. SPN)	79 (22 NF-pNETs, 57 SPNs)	RW (TS:DS = 7:3)	Histopathology	T2WI, DWI, T1WI, CE-T1WI	Precontrast T1WI: AUC 0.853 AP: AUC 0.907 PVP: AUC 0.773 DP: AUC 0.773 Clinic-radiomics nomogram: AUC 0.920, Acc 90.0%, Sen 100.0%, Spec 71.4%
Li	2019	MaZda (v 4.6)	Differential diagnosis (NF-pNET vs. SPN)	119 (61 NF-pNETs, 58 SPNs)	RW (101 TS, 18 DS)	Histopathology	T2WI, DWI, T1WI, CE-T1WI	AP: AUC 0.925 DP: AUC 0.950
Cui	2021	MITK Software (v 3.1.0.A)	IPMN grading	202 (152 low-grade, 50 high-grade)	RW (103 TS, 48 VS1, 51 VS2)	Histopathology	T2WI, T1WI, CE-T1WI	SET 1 Radiomics signature: AUC 0.811; Nomogram: AUC 0.884, Sen 90.0%, Spe 79.0% SET 2 Radiomics signature: AUC 0.822; Nomogram: AUC 0.876, Sen 85.7%, Spe 83.7%
Jeon	2021	MEDIP	Prediction of IPMN malignancy	248 (142 Benign, 106 Malignant)	SW	Histopathology	MRCP	AUC 0.85 (Greater entropy and smaller compactness as independent predictors)
Guo	2019	Omni-Kinetics software (v 2.0.10)	pNET grading	77 (31 grade 1, 29 grade 2, 17 grade 3)	RW	Histopathology	T2WI, DWI, T1WI, CE-T1WI	Independent predictors of T2WI: inverse difference moment for G1 vs. G2 (AUC 0.833), energy+correlation+difference entropy for G1 vs. G3 (AUC 0.989), difference entropy for G2 vs. G3 (AUC 0.813); Independent predictors of DWI: correlation+contrast+inverse difference moment for G1 vs. G2 (AUC 0.841), maxintensity+entropy+inverse difference moment for G1 vs. G3 (AUC 0.962), maxintensity for G2 vs. G3 (AUC 0.703)
Kaissis	2019	Pyradiomics	PDAC prognosis	132	SW (100 TS, 32 VS)	Histopathology	T2WI, DWI, T1WI, CE-T1WI	AUC 0.90, Sen 87%, Spe 80%
Kaissis (1)	2019	Pyradiomics	PDAC classification	55 (27 KRT81+, 28 KRT81-)	SW	Histopathology	T2WI, DWI, T1WI, CE-T1WI	AUC 0.93, Sen 90%, Spe 92%
Taffel	2019	In-house software FireVoxel	Tumor diagnosis	42 (36 PDACs, 6 pNETs)	SW	Histopathology	T2WI, DWI, T1WI, CE-T1WI	ADC histogram differentiation NET-PDAC: AUC 0.88-0.92, Sen 94–97%, Spe 83–88%; Differentiation nodal status: AUC 0.80–0.82, Sen 87%, Spe 67–83%
Becker	2017	In-house algorithm (MATLAB R2015b)	Impact of b-values	8 controls	RW	Radiologist	DWI	Significant positive correlations with b-value: skewness, contrast, correlation, energy, LRE, GLN, RP; Significant negative correlations with b-value: kurtosis, entropy, homogeneity, LGRE, SRLGE, LRLGE
Lin	2019	IBEX	AcP classification	259 (142 mild AcP, 117 severe AcP)	SW (180 TS, 79 VS)	Radiologist	CE-T1WI	AUC 0.848, Acc 81.0%, Sen 75.0%, Spe 86.0%
Frokjaer	2020	SlicerRadiomics extension (v 4.10.1)	CP classification	99 (77 CP, 22 controls)	SW	Radiologist	T2WI, DWI, MRCP	Acc 98%, Sen 97%, Spe 100%

Acc—accuracy, ADC—apparent diffusion coefficient, AP—arterial phase, AcP—acute pancreatitis, AUC—area under the curve, CE—contrast-enhanced, CP—chronic pancreatitis, DP—delayed phase, DWI—diffusion-weighted imaging, IPMN—intraductal papillary mucinous neoplasm, MRCP—magnetic resonance cholangiopancreatography, MRI—magnetic resonance imaging, PDAC—pancreatic ductal adenocarcinoma, pNET—pancreatic neuroendocrine tumor, NF-pNET—nonfunctioning pancreatic neuroendocrine tumor, PVP—portal venous phase, RW—record-wise, Sen—sensitivity, Spe—specificity, SPN—solid pseudopapillary neoplasm, SW—subject-wise, T1WI—T1-weighted imaging, T2WI—T2-weighted imaging, TS—training set, VS—validation set.

**Table 4:** Applications of radiomics in pancreatic PET-MRI images.

Author	Year	Radiomics Analysis	Task	N Pts	Data Split	Reference Standard	Radiotracer	MRI Phase	Results
Gao	2020	LIFEx software	Prediction of metastatic disease	17 (11 metastatic PDACs, 6 non-metastatic PDACs)	RW	Radiologist and nuclear medicine physician	FDG	T2W HASTE, DWI, T1WI DIXON	SUV: AUC 0.818, Sen 72.7%, Spe 100%MTV: AUC 0.818, Sen 63.6%, Spe 100%TLG: AUC 0.848, Sen 72.7%, Spe 100%

AUC—area under the curve, DWI—diffusion-weighted imaging, FDG—fluorodeoxyglucose, HASTE—half-Fourier acquisition single-shot turbo spin-echo sequence, MRI—magnetic resonance imaging, MTV—metabolic tumor volume, PDAC—pancreatic ductal adenocarcinoma, RW—record-wise, Sen—sensitivity, Spe—specificity, SUV—standardized uptake value, T1WI—T1-weighted imaging, T2WI—T2-weighted imaging, TLG—total lesion glycolysis.

**Table 5:** Applications of radiomics in pancreatic CT and MRI images.

Author	Year	Radiomics Analysis	Task	N Pts	Data Split	Reference Standard	CT/MRI Phase	Results
Azoulay	2019	TexRAD	Differential diagnosis (G3-pNET vs. NEC)	37 (14 G3-pNETs, 23 NECs)	RW	Radiologist	CT: NECT, AP, PVP MRI: T1WI, T2WI, DWI, AP, PVP	CT histogram analysis AP skewness filter 4: AUC 0.736 AP skewness filter 5: AUC 0.758 PVP mean filter 0:AUC 0.712 PVP MPP filter 0: AUC 0.712 PVP entropy filter 0: AUC 0.719
Ohki	2021	NS	pNET Grading (G1 vs. G2–G3)	33 (22 grade 1, 11 grade 2/3)	RW	Radiologist	CT: AP, PVP MRI: ADC map	AP log-sigma 1.0 joint-energy: AUC 0.855 PVP log-sigma 1.5 kurtosis: AUC 0.860 ADC log-sigma 1.0 correlation: AUC 0.847

AP—arterial phase, AUC—area under the curve, CT—computed tomography, DWI—diffusion-weighted imaging, MPP—mean of positive pixels, MRI—magnetic resonance imaging, NEC—neuroendocrine carcinoma, NECT—non-enhanced CT, NS—not specified, pNET—pancreatic neuroendocrine tumor, PVP—portal venous phase, RW—record-wise, T1WI—T1-weighted imaging, T2WI—T2-weighted imaging.

**Table 6:** Applications of CAD in pancreatic CT images.

Author	Year	AI Model	Task	N Pts	Data Split	Reference Standard	CT Phase	Results
Li	2016	SVM	Differential diagnosis (SOA vs. MCN)	42 (23 SOAs, 19 MCNs)	RW	Radiologist	NECT, AP, PVP	Acc 93.2%
Liu	2020	CNN	Tumor diagnosis	690 local set 1(370 cases, 320 controls), 189 local set 2 (101 cases, 88 controls), 363 US test set (281 cases, 82 controls)	SW (412 TS, 139 VS, 139 test set 1, 189 test set 2)	Pathology	PVP	Local set 1: AUC 0.997, Acc 98.6%, Sen 97.3%, Spe 100% Local set 2: AUC 0.999, Acc 98.9%, Sen 99.0%, Spe 98.9% US set: AUC 0.920, Acc 83.2%, Sen 79.0%, Spe 97.6%
Roy	2020	ANN	Tumor segmentation	NS	NS	NS	NS	NS
Gibson	2018	Dense V-Network FCN	Pancreas segmentation	90 (43 public dataset 1, 47 public dataset 2)	SW	Radiologist	CECT	DSC 78%
Xue	2021	3D FCN	Pancreas segmentation	59	SW	Radiologist	CECT	DSC 86.9% JC 77.3%
Zheng	2020	VNet	Pancreas segmentation	82	RW	Radiologist	CECT	DSC 86.21% Sen 87.49% Spe 85.11%
Boers	2020	Interactive 3D UNet	Pancreas segmentation	100	RW (90 TS, 10 VS)	Radiologist	PVP	DSC 78.1%, average automated baseline performance 78%, semiautomatic segmentation performance in 8 min 86%
Suman	2021	NVIDIA	Pancreas segmentation	188 first batch, 159 second batch	SW	Radiologist	PVP	DSC 63%, JC 48%, FP 21%, FN 43%
Nishio	2020	Deep UNet	Pancreas segmentation	80	RW	Radiologist	CECT	DSC 70.3–78.9%, JC 0.563–0.658, Sen 64.5–76.2%, Spe 100%
Panda	2021	3D CNN	Pancreas segmentation	1917 internal dataset, 41 external dataset 1, 80 external dataset 2	RW (1380 TS, 248 VS, 289 internal test set, 50 external test set 1, 82 external test set 2)	Radiologist	PVP	Internal dataset: DSC 91% External dataset 1: DSC 83–84% External dataset 2: DSC 89%
Li	2021	MAD-UNet	Pancreas segmentation	363 (82 public dataset 1, 281 public dataset 2)	RW	UNet, VNet, Attention UNet, SegNet	CECT	DSC 86.10% JC 75.55% Sen 86.43% Spe 84.97%

Acc—accuracy, ANN—artificial neural network, AP—arterial phase, AUC—area under the curve, CECT—contrast-enhanced computed tomography, CNN—convolutional neural network, CT—computed tomography, DSC—Dice similarity coefficient, FCN—fully convolutional network, FN—false negative, FP—false positive, JC—Jaccard coefficient, MCN—mucinous cystic neoplasm, NECT—non-enhanced computed tomography, NS—not specified, PVP—portal venous phase, RW—record-wise, Sen—sensitivity, SOA—serous oligocystic adenoma, Spe—specificity, SW—subject-wise, TS—training set, VS—validation set.

**Table 7:** Applications of CAD in pancreatic PET-CT images.

Author	Year	AI Model	Task	N Pts	Data Split	Reference Standard	Radiotracer	CT Phase	Results
Li	2018	HFB-SVM-RF	Tumor Diagnosis	80 (40 cancer patients, 40 controls)	RW	Radiologist	FDG	NECT	Acc 96.47%, Sen 95.23%, Spe 97.51%

Acc—accuracy, AI—artificial intelligence, CT—computed tomography, FDG—fluorodeoxyglucose, HFB-SVM-RF—hybrid feedback-support vector machine-random forest, NECT—non-enhanced computed tomography, RW—record-wise, Sen—sensitivity, Spe—specificity.

**Table 8:** Applications of CAD in pancreatic MRI images.

Author	Year	AI Model	Task	N Pts	Data Split	Reference Standard	MRI Phase	Results
D’Onofrio	2021	NS	Prediction of IPMN malignancy	91	SW	Histopathology	T2WI, T1WI, DWI, MRCP	ADC map: entropy = 10.32, J Youden index 0.48, AUC 0.7288, Sen 68.75%, Spe 79.25%
Balasubramanian	2019	ANN, SVM	Tumor diagnosis	168 (68 with lesion, 100 controls)	RW (TS:VS = 7:3)	NS	NS	ANN BP 2 features (HOMO, CP): Acc 98%, Sen 100%, Spe 95%
Barbieri	2020	DNN	Evaluation of IVIM performance	10	SW	Radiologist	DWI	Dt: ICC 94–97% Fp: ICC 66% Dp: 50–51%
Chen	2020	UNet-based ALAMO	Pancreas segmentation	102	SW (66 TS, 16 VS, 20 test set)	Radiologist	T1WI-VIBE	Single slice: DSC 0.871 20 slices: DSC 0.880 40 slices: DSC 0.871

Acc—accuracy, ADC—apparent diffusion coefficient, ALAMO—automated deep learning-based abdominal multiorgan segmentation, ANN—artificial neural network, AUC—area under the curve, CP—cluster prominence, Dp—pseudo-diffusion coefficient, DSC—Dice similarity coefficient, Dt—pure diffusion coefficient, DWI—diffusion-weighted imaging, Fp—perfusion fraction, HOMO—homogeneity, IVIM—intravoxel incoherent motion, MRCP—magnetic resonance cholangiopancreatography, MRI—magnetic resonance imaging, NS—not specified, RW—record-wise, Sen—sensitivity, Spe—specificity, SVM—support vector machine, SW—subject-wise, T1WI—T1-weighted imaging, T2WI—T2-weighted imaging, TS—training set, VIBE—volumetric interpolated breath-hold examination, VS—validation set.

## Data Availability

No new data were created or analyzed in this study. Data sharing does not apply to this article.

## Supplementary Materials

The following supporting information can be downloaded at: https://www.mdpi.com/article/10.3390/healthcare10081511/s1, File S1: Search strategy.
