# Supplementary material for: Artificial Intelligence Applied to Pancreatic Imaging: A Narrative Review
Source: Healthcare (Basel). 2022 Aug 11;10(8):1511. doi: 10.3390/healthcare10081511 (PMC9408381; doi:10.3390/healthcare10081511)
Supplement: Supplementary file 1 [file healthcare-10-01511-s001.zip › healthcare-1803216-supplementary.pdf]

Supplementary Material File S1. Search strategy

We performed a systematic search of PubMed/Medline, Embase, Web of Science, Cochrane library and Scopus for articles relevant to application of AI and radiomics in pancreatic imaging.

The search algorithm was constructed using the following strings for each library:

PubMed:

Query: ("artificial intelligence"[MeSH Terms] OR ("artificial"[All Fields] AND "intelligence"[All Fields]) OR "artificial intelligence"[All Fields] OR "Deep Learning"[MeSH Terms] OR "Deep Learning"[All Fields] OR "Machine Learning"[MeSH Terms] OR "Machine Learning"[All Fields] OR "neural networks, computer"[MeSH Terms] OR "Neural Network"[All Fields] OR ("radiomic"[All Fields] OR "radiomics"[All Fields]) OR "Texture analysis"[All Fields]) AND (pancrea* AND ("segmentation" OR "neoplasm" OR "cancer" OR "tumor" OR "lesion") OR "pancreatitis")

SCOPUS:

Query: TITLE-ABS-KEY (("artificial intelligence" OR "Deep Learning" OR "Machine Learning" OR "neural networks, computer" OR "Neural Network" OR radiomic OR "Texture analysis") AND (("pancrea*" AND ("segmentation" OR "neoplasm" OR "cancer" OR "tumor" OR "lesion")) OR "pancreatitis"))

Embase

Query: ('artificial intelligence'/exp OR 'artificial intelligence' OR 'artificial intelligence':ab,ti OR 'deep learning'/exp OR 'deep learning' OR 'deep learning':ab,ti OR 'machine learning'/exp OR 'machine learning' OR 'machine learning':ab,ti OR 'neural network'/exp OR 'neural network' OR 'neural network':ab,ti OR radiomic:ab,ti OR 'texture analysis':ab,ti) AND (('pancreas'/exp OR 'pancrea*') AND (segmentation OR neoplasm OR 'cancer'/exp OR tumor OR lesion) OR 'pancreatitis':ab,ti)

WoS Core Collection:

Query: TOPIC: (("artificial intelligence" OR "Deep Learning" OR "Machine Learning" OR "neural networks, computer" OR "Neural Network" OR radiomic OR "Texture analysis") AND (pancrea* AND ("segmentation" OR "neoplasm" OR "cancer" OR "tumor" OR "lesion") OR "pancreatitis"))

Cochrane

Query in Title Abstract Keyword: (artificial intelligence OR machine learning OR deep learning OR neural network OR radiomic OR Texture analysis) AND (pancrea* AND (segmentation OR neoplasm OR cancer OR tumor OR lesion) OR pancreatitis)
